# Advancements in diabetic foot insoles: a comprehensive review of design, manufacturing, and performance evaluation

**DOI:** 10.3389/fbioe.2024.1394758

**Published:** 2024-07-15

**Authors:** Yuanfei Ren, Hao Wang, Xiaoshuang Song, Yanli Wu, Yongtao Lyu, Wei Zeng

**Affiliations:** ^1^ The First Department of Hand and Foot Surgery, Central Hospital of Dalian University of Technology, Dalian, China; ^2^ Department of Engineering Mechanics, School of Mechanics and Aerospace Engineering, Dalian University of Technology, Dalian, China; ^3^ DUT-BSU Joint Institute, Dalian University of Technology, Dalian, China; ^4^ Department of Mechanical Engineering, New York Institute of Technology, New York, NY, United States

**Keywords:** diabetic foot, customized insoles, finite element analysis, pressure offloading, additive manufacturing, smart insoles

## Abstract

The escalating prevalence of diabetes has accentuated the significance of addressing the associated diabetic foot problem as a major public health concern. Effectively offloading plantar pressure stands out as a crucial factor in preventing diabetic foot complications. This review comprehensively examines the design, manufacturing, and evaluation strategies employed in the development of diabetic foot insoles. Furthermore, it offers innovative insights and guidance for enhancing their performance and facilitating clinical applications. Insoles designed with total contact customization, utilizing softer and highly absorbent materials, as well as incorporating elliptical porous structures or triply periodic minimal surface structures, prove to be more adept at preventing diabetic foot complications. Fused Deposition Modeling is commonly employed for manufacturing; however, due to limitations in printing complex structures, Selective Laser Sintering is recommended for intricate insole designs. Preceding clinical implementation, *in silico* and *in vitro* testing methodologies play a crucial role in thoroughly evaluating the pressure-offloading efficacy of these insoles. Future research directions include advancing inverse design through machine learning, exploring topology optimization for lightweight solutions, integrating flexible sensor configurations, and innovating new skin-like materials tailored for diabetic foot insoles. These endeavors aim to further propel the development and effectiveness of diabetic foot management strategies. Future research avenues should explore inverse design methodologies based on machine learning, topology optimization for lightweight structures, the integration of flexible sensors, and the development of novel skin-like materials specifically tailored for diabetic foot insoles. Advancements in these areas hold promise for further enhancing the effectiveness and applicability of diabetic foot prevention measures.

## 1 Introduction

Diabetic foot represents a severe condition characterized by infection, ulceration, or tissue destruction in the foot of individuals with diabetes, often concomitant with lower extremity neuropathy, peripheral artery disease (particularly lower extremity arterial disease) (Jodheea-Jutton et al., 2022), or Charcot foot. According to the comprehensive analysis conducted by Zhang et al. (2017), the aggregated global prevalence of diabetic foot ulceration stands at 6.3% (95% confidence intervals: 5.4%–7.3%). This prevalence is on a continual rise, mirroring the escalating numbers of individuals afflicted by diabetes and those at risk, thereby establishing the diabetic foot as a burgeoning global public health challenge (van Netten et al., 2020; Edmonds et al., 2021).

The surge in diabetic foot cases has intensified the demand for effective prevention and treatment strategies. Diabetic foot ulcers (DFU), infections, or soft tissue damage often evade early detection, particularly in neuropathic feet, as the typical indicators of local infection, such as redness, pain, or tenderness, may be absent (Edmonds et al., 2021). Complicating matters, diabetic patients with deep foot infections may not exhibit traditional signs like an elevated white blood cell count and temperature, leading to delayed diagnosis and treatment (Apelqvist, 2012; Yu et al., 2022). Common imaging tests prove effective primarily in diagnosing diabetic foot conditions in the presence of bone alterations, abscess formation, and soft tissue destruction, missing the optimal window for intervention (Senneville et al., 2020; Yufeng and Yanwu, 2021).

Treatment and care pose challenges, including intricate cures, high recurrence rates, and elevated treatment costs (Kerr et al., 2019; Edmonds et al., 2021). The 5-year mortality and direct care costs for patients with diabetic foot complications rival those of cancer (Armstrong et al., 2020). Lack of awareness about prevention further compounds the issue, making treatment more challenging once diagnosed. In the initial stages of diabetic foot, lower limb pain during walking may be alleviated or disappear after resting, leading high-risk groups to neglect prevention services, increasing the likelihood of severe ulcers or amputations (Chatwin et al., 2020). Therefore, early prevention and intervention during the initial stages of disease development are crucial for diabetic foot patients (Lim et al., 2017). The utilization of specialized therapeutic insoles emerges as a commendable preventive measure (Schaper et al., 2020). These insoles are widely employed to mitigate plantar pressure peaks or optimize pressure distribution, effectively reducing the likelihood of complications and amputations (Korada et al., 2020). Nevertheless, challenges persist in further optimizing pressure offloading performance, enhancing comfort, and streamlining the design and manufacturing cycle and cost of customized insoles (Collings et al., 2021).

**FIGURE 1:**
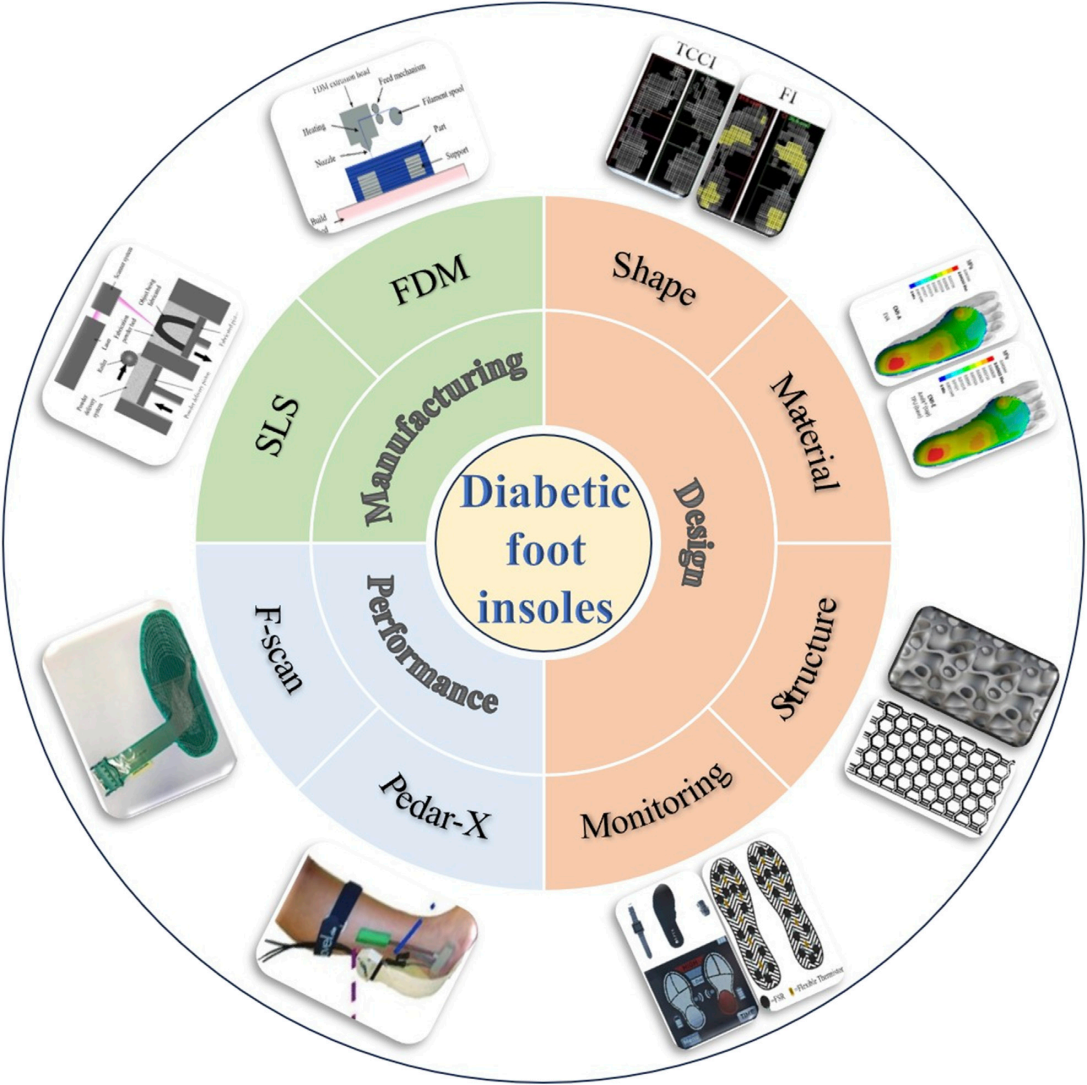
Schematic diagram of design, manufacture, and evaluation of diabetic foot insoles (D’Amico et al., 2021; Nouman et al., 2021; Mondal et al., 2023; Claybrook et al., 2022; Khandakar et al., 2022; Abbott et al., 2019; Chatwin et al., 2020; Aliberti et al., 2011; Alafaghani et al., 2017; Abdulhameed et al., 2019).

In the realm of diabetic foot insoles, notable advancements in design approaches and manufacturing techniques have brought about significant transformations, leading to a marked improvement in their performance, as illustrated in Figure 1. The triad of design, manufacturing, and performance evaluation stands as a critical nexus within the domain of diabetic foot insoles, with each facet exerting a profound influence on the others. Advanced manufacturing technologies now facilitate the creation of diabetic foot insoles boasting intricate structures, including honeycomb porous configurations and triply periodic minimal surface (TPMS) structures. These elaborate structures encounter challenges in precision and production costs when subjected to conventional manufacturing methods such as hand forming or numerical control techniques. Concurrently, the evaluation of diabetic foot insole performance serves as a driving force behind advancements in manufacturing and design. Thus, the current study offers a comprehensive review of the design, manufacturing, and performance evaluation of diabetic foot insoles, aiming to propel further breakthroughs in this dynamic field.

## 2 Review methods

### 2.1 Literature search strategy and selection criteria

We conducted searches in English-language databases, including PubMed, Web of Science, Elsevier ScienceDirect, SpringerLink, and Wiley Online Library. The search terms used were “diabetic foot” OR “"diabetic feet” OR “diabetic foot ulcer” OR “diabetic foot problem” AND “insoles” OR “smart insoles” OR “orthotic”; “diabetic foot insoles” OR “diabetic foot ulcer” AND “design” OR “biomechanics” OR “finite element” OR “pressure offloading”; “diabetic foot insoles” OR “diabetic foot ulcer” AND “manufacturing” OR “additive manufacturing”; “diabetic foot insoles” AND “evaluation”. We did not restrict the study design or the geographic level (national or regional) of the studies. All databases were searched from January 2000 to January 2023. We also reviewed the references of all included articles to identify other potentially relevant surveys.

**FIGURE 2:**
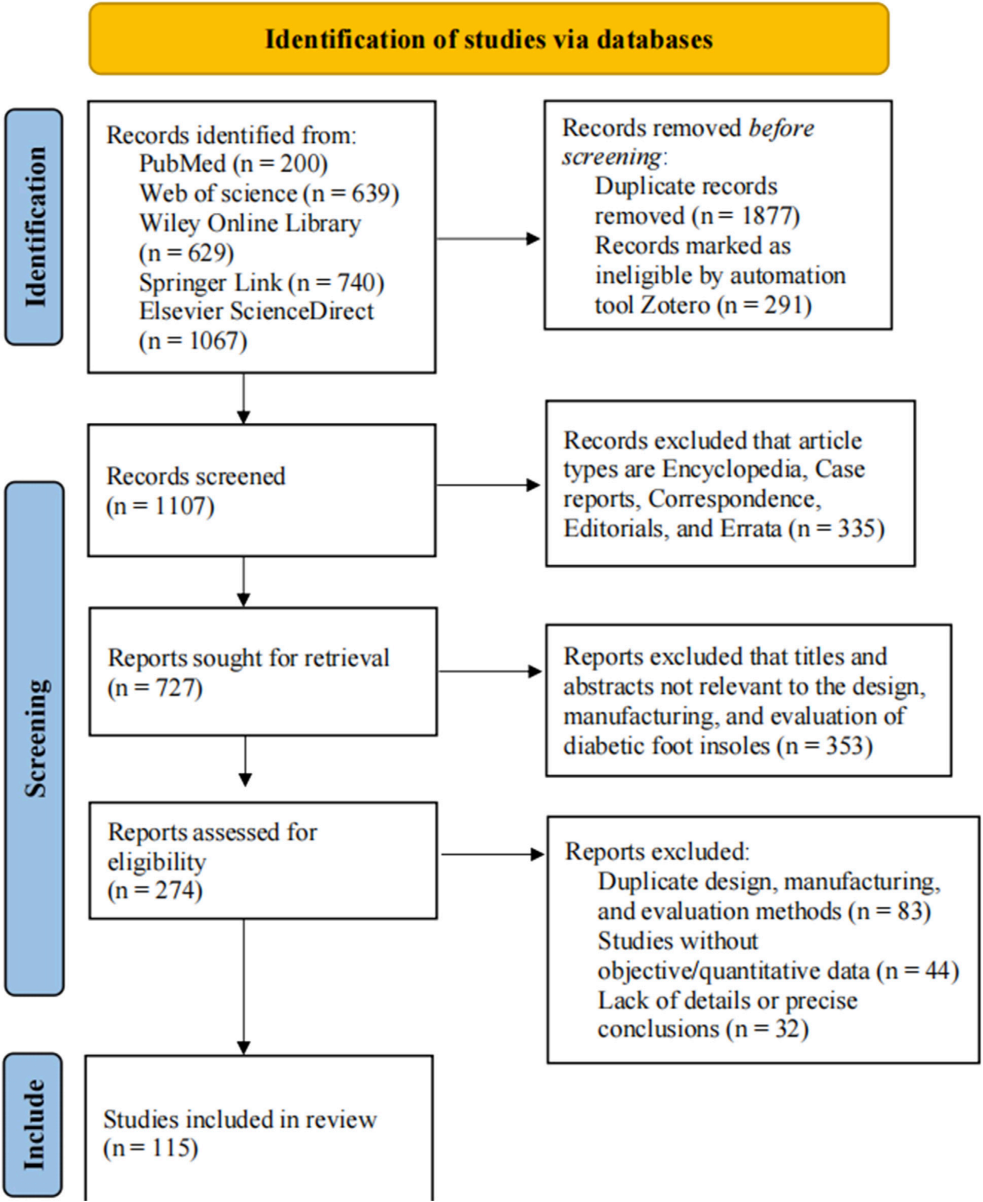
Identification of studies via databases.

The study inclusion criteria were as follows: firstly, the data needed to be presented in English; secondly, detailed methodology for the design, manufacturing, and evaluation of diabetic foot insoles needed to be described; thirdly, the study had to provide sufficient information to enable the design of diabetic foot smart insoles. If multiple articles were based on the same methodology, only the study with the most complete data was included. Furthermore, abstracts meeting the inclusion criteria were also included. The study exclusion criteria were as follows: firstly, duplicate methodologies needed to be excluded; secondly, studies without objective or quantitative data needed to be excluded; finally, studies lacking design and analysis details, or accurate conclusions needed to be excluded. The PRISMA flow diagram of identifying studies via databases is shown in Figure 2.

In case of disagreement during the study selection process, we followed pre-established inclusion and exclusion criteria to minimize subjectivity and improve consistency. If consensus could not be reached, we consulted relevant experts. Inclusion and exclusion criteria were reconsidered if necessary to ensure appropriateness and consensus for final selection.

### 2.2 Results of searching

The number of studies included about 18 studies on the topic of “Introduction”; about 62 studies on the topic of “Design of Diabetic Foot Insoles”; about 26 studies on the topic of “Additive Manufacturing of Diabetic Foot Insoles”; and about 21 studies on the topic of “Performance Evaluation of Diabetic Foot Insoles”. There were 12 studies that contained multiple topics, so they were included twice. Among the general participants in these included studies, researchers in Europe and North America were more active than those in other regions. Some major research centers and laboratories are located in countries such as the US, UK, Germany, and the Netherlands. Additionally, many researchers included in the studies are affiliated with international organizations and associations, such as the International Diabetic Foot Association and the European Association for the Study of Diabetes Foot.

## 3 Design of diabetic foot insoles

**FIGURE 3:**
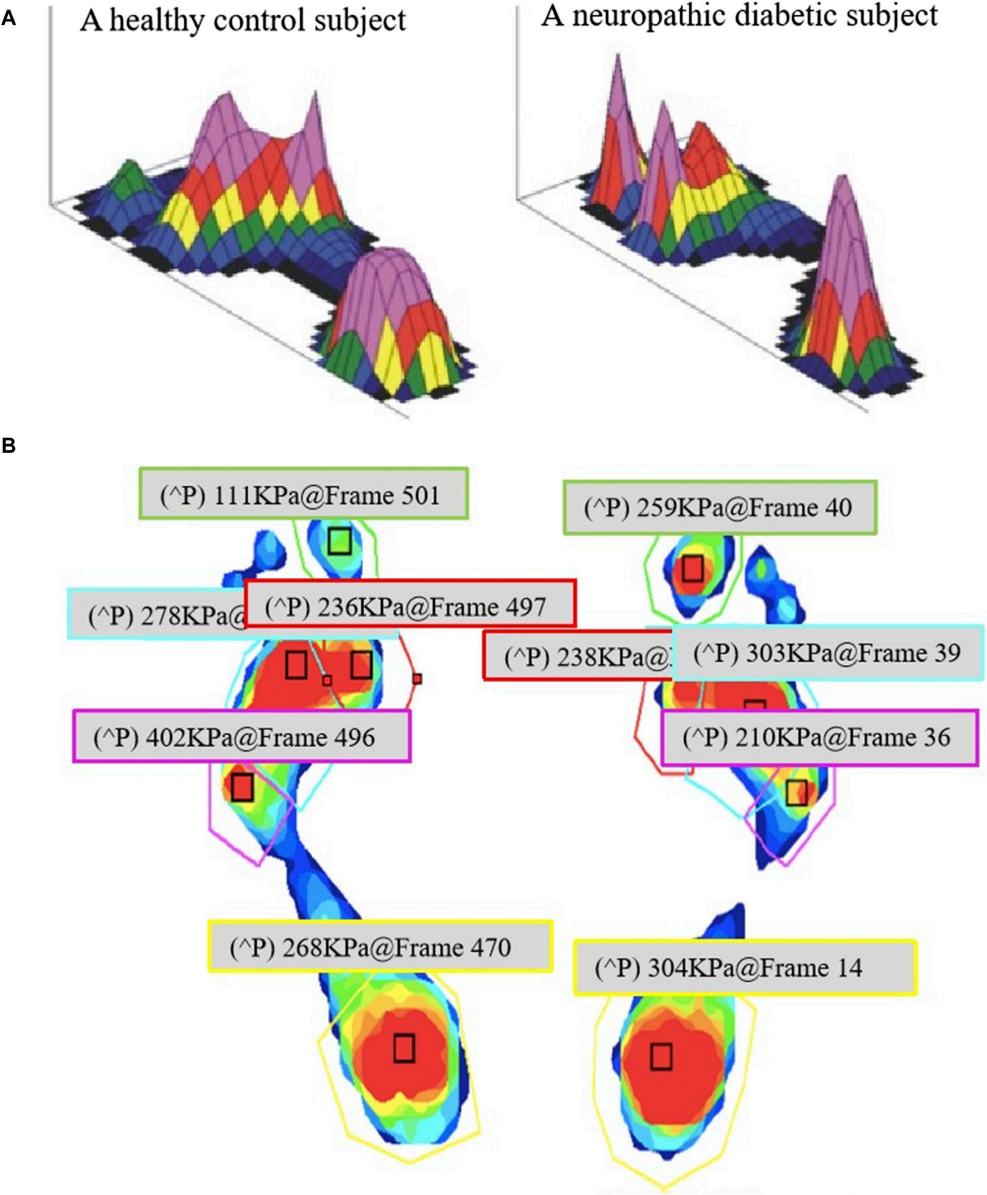
Comparison of the plantar pressure: **(A)** Comparison of peak plantar pressure in a healthy subject and a neuropathic diabetic subject (Bus et al., 2021), and **(B)** Different regions with different plantar pressures (Perren et al., 2021).

The development of diabetic foot is not solely linked to neuropathy and changes in blood glucose but is also influenced by biomechanics (Asghar and Naaz, 2022). The plantar pressure distribution in healthy individuals versus diabetic foot patients differs significantly, as depicted in Figure 3A (Deschamps et al., 2013; Fernando et al., 2016; Bus et al., 2021). Moreover, variations exist in pressure conditions across different plantar areas, as illustrated in Figure 3B (Perren et al., 2021). Ground pressure, excessive plantar shear stress (Jones et al., 2022), and compressive stress from shoes or adjacent toes collectively impact diabetic foot development (Allan et al., 2016). Designing specialized insoles for diabetic foot patients becomes imperative due to these complexities. Diabetic foot insoles can be broadly categorized into two groups: pressure offloading insoles and smart monitoring insoles.

### 3.1 Design of pressure offloading diabetic foot insoles

Local lesions exacerbate foot ulcers when certain sole parts are over-pressed (Singh et al., 2021). Therefore, offloading plantar pressure or optimizing pressure distribution effectively prevents and treats diabetic foot (Bellomo et al., 2022). Enhancing the pressure offloading performance involves optimizing the insole’s shape, material, and structure (Haris et al., 2021). Additionally, the comfort levels of insoles with different shapes, materials, and structures vary. Considering that diabetic patients may need to wear insoles for extended periods, discomfort significantly affects their regular usage, potentially compromising prevention or treatment effectiveness.

**FIGURE 4:**
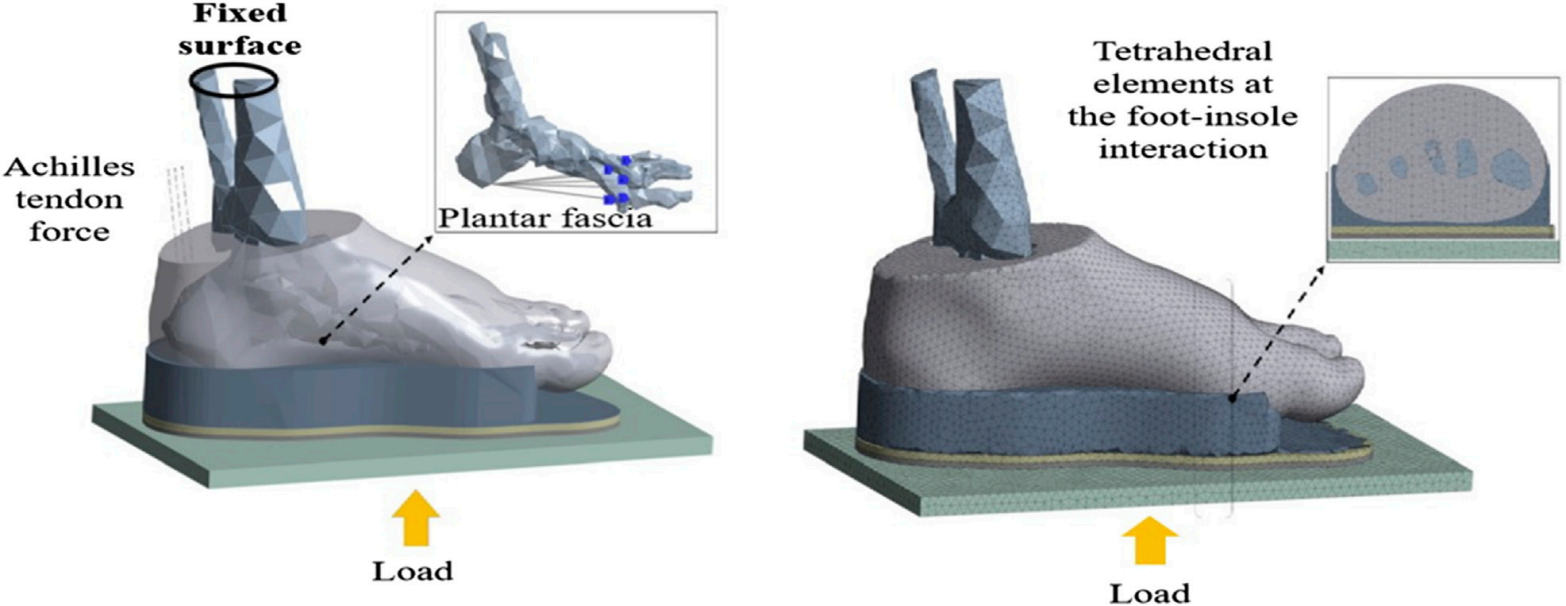
The FE foot model with customized insole and ground (Nouman et al., 2023).

The finite element (FE) method has emerged as a key tool in designing diabetic foot insoles (Moayedi et al., 2021). These simulations aim to enhance our understanding of foot mechanics in health and disease, guiding the design of personalized insoles (Erdemir et al., 2012). The FE model for insole design is depicted in Figure 4. Through finite element analysis (FEA) (Zeng et al., 2021), we can predict the pressure offloading performance of insoles with different shapes, materials, and structures, optimizing their design for improved diabetic foot insole performance (Ghassemi et al., 2015). However, these simulations have limitations, and future research needs to consider additional biomechanical variables. Complex processes involving the interaction and response of various foot tissues, such as bones, muscles, and fascia, necessitate the consideration of biomechanical variables like joint angles, moments, muscle activity, skeletal stress, and strain for a comprehensive evaluation of foot motion, stability, muscle coordination, functionality, and the prediction of foot disease risks.

#### 3.1.1 Shape of the diabetic foot insole

**FIGURE 5:**
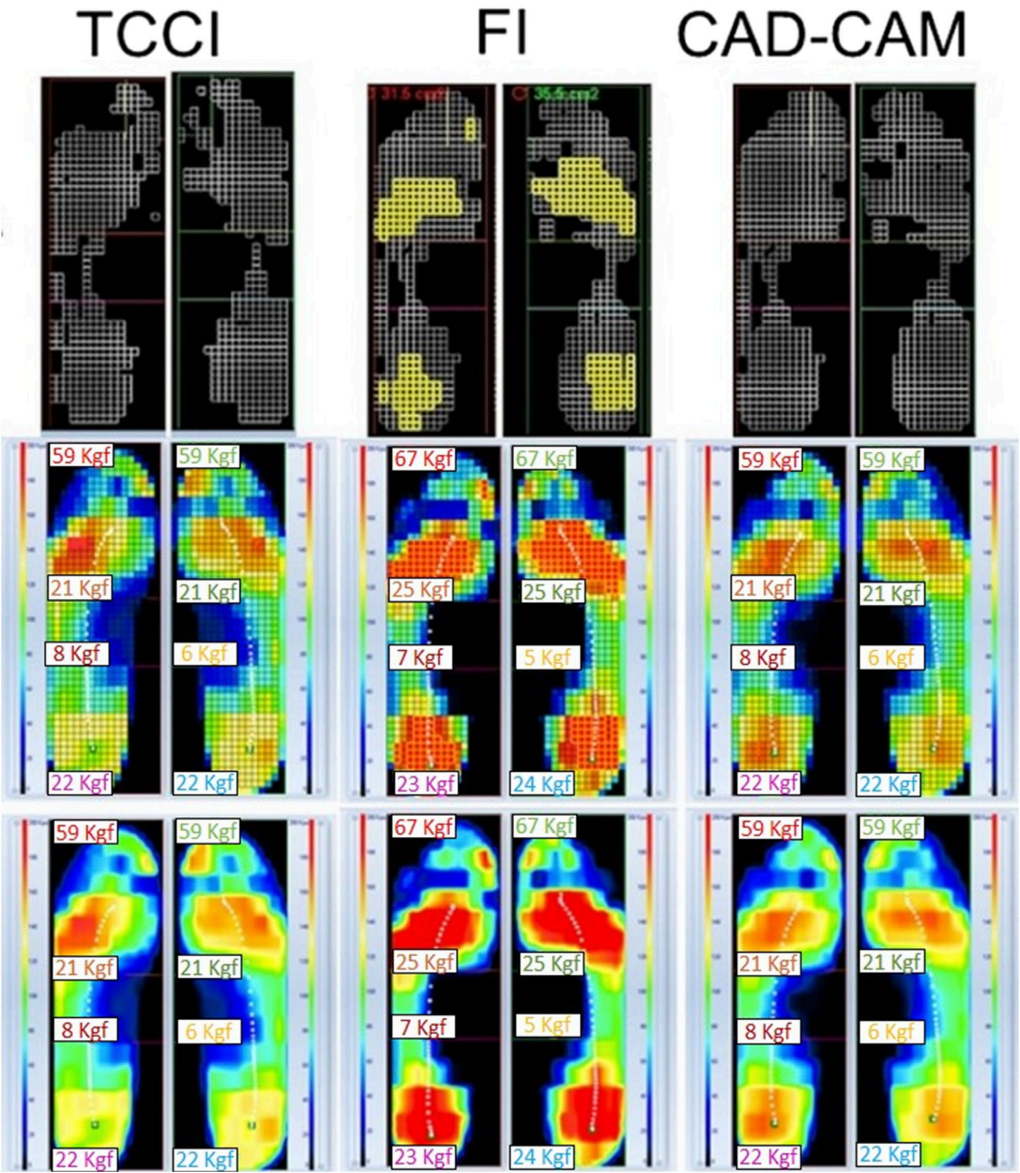
Comparison of pressure redistribution effects of three different shapes of insoles–flat insoles (FI), total contact customized insoles (TCCI), Computer Aided Design-Computer Aided Manufacturing (CAD-CAM) insoles (D’Amico et al., 2021).

In designing the insole, determining the insole’s shape is crucial. Due to individual foot differences, custom-molded shapes are critical for reducing peak plantar pressure in diabetic foot insoles (Martinez-Santos et al., 2019). Jafarzadeh et al. (2021) optimized insole shape using Python and MATLAB programming, starting from a 25.0 mm flat insole. ABAQUS was then used for contact pressure analysis, revealing a 25% reduction in maximum plantar pressure compared to the flat insole. Studies indicate that total contact insoles offer more benefits to diabetic foot than flat insoles (Ahmed et al., 2020; Shim et al., 2021). El-Hilaly et al. (2013) found the efficacy of total contact insoles in offloading foot pressures in diabetic patients following partial first-ray amputations, leading to significant pressure reduction compared to flat insoles during standing and walking. D’Amico et al. (2021) compared Computer Aided Design-Computer Aided Manufacturing (CAD-CAM) and traditional total contact customized insoles (TCCI), showing both reduced risk areas and optimized plantar pressure distribution compared to flat insoles (Figure 5). Arch-supported insoles were found beneficial for diabetic feet with a high arch, while total contact insoles were less favorable for flat feet (Niu et al., 2020). Table 1 summarizes studies on the shape of diabetic foot insoles. Multidisciplinary design methods incorporating computational and mathematical approaches have been explored for insole design. Shalamberidze et al. (2021) applied integral curves of solutions to Dirichlet singular boundary differential equations (Rachůnková et al., 2013) to accurately describe the geometric shapes of overpressure regions. A computer program based on this mathematical algorithm designed individualized orthopedic insoles, offering new avenues for future research.

**TABLE 1:** Key literature on the shape of diabetic foot insoles.

Representative study	Shape	Method	Main findings and conclusion
Jafarzadeh et al. (2021)	Optimized insole shape based on a flat insole	Using Python and Matlab programming	The maximum plantar pressure decreased by 25% compared to the flat insole
El-Hilaly et al. (2013)	Total contact insole	Measuring plantar pressure	Showing a 49% pressure change when standing, 54% change in the M3 area, and only 27% change when walking with the flat insole
D’Amico et al. (2021)	CAD-CAM total contact customized insoles	Personalized foot data-driven design	The CAD-CAM approach performed better than the TCCI^a^ with a mean pressure reduction of 37.3 kPa (15.6%) vs. FI
Niu et al. (2020)	arch-supported insole	Finite element analysis	Reducing the peak pressure from 208.86 to 160.02 kPa; be beneficial to diabetic foot with a high arch, but unfavorable to flat feet

^a^ TCCI: total contact customized insoles, M3: mid metatarsal area.

#### 3.1.2 Material of the diabetic foot insole

**FIGURE 6:**
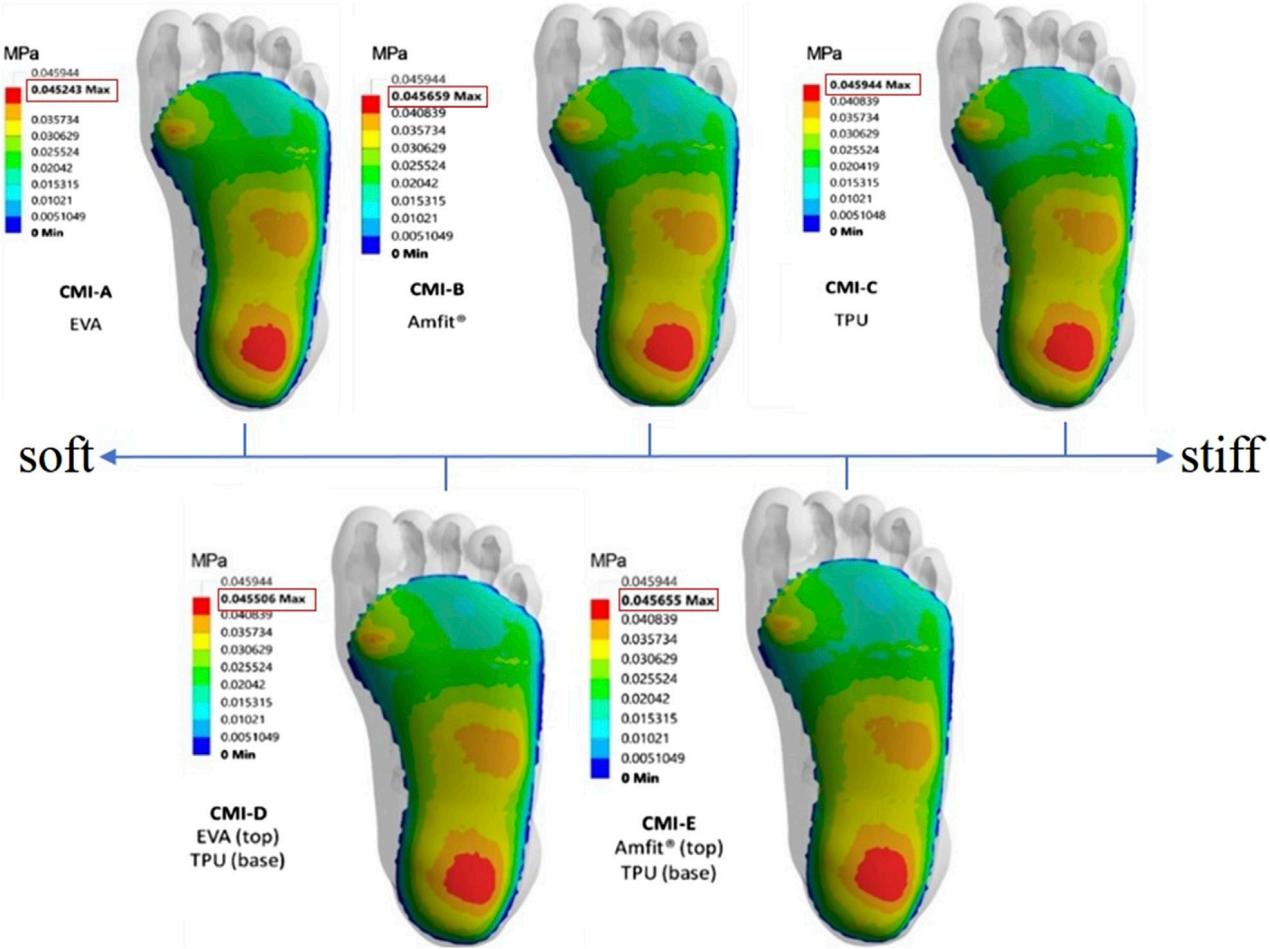
The contact pressure (MPa) distribution with soft material to stiffer material and its combinations (Nouman et al., 2021).

Mechanical property differences among materials affect the pressure offloading performance of customized insoles (Nouman et al., 2019; Haris et al., 2021). Telfer et al. (2014) identified footwear material elasticity as the most influential factor in pressure offloading. Studies comparing pressure offloading performance across different insole materials (Ma et al., 2019; Nouman et al., 2019; Tang et al., 2019; Chhikara et al., 2022) provide practical information for clinical prevention, guiding the design of more suitable insoles for diabetic patients. Soft insole materials, such as PORON Medical 4,708 and Nora Lunalastik EVA, outperformed rigid materials like Nora Lunalight A fresh and Pe-Lite in plantar pressure offloading for diabetic elderly individuals (Shi et al., 2022). Additionally, uniform softening of a homogeneous insole led to a 30% decrease in peak plantar pressure (Jafarzadeh et al., 2021). Nouman et al. (2021) found that a combination of soft and hard materials achieved a more uniform distribution of contact pressure (Figure 6). Table 2 provides a comparison of materials used in diabetic foot insoles.

**TABLE 2:** Comparison of materials for diabetic foot insoles.

Materials for diabetic foot insoles		Characteristics	Refs
Soft material	PORON Medical 4708	Be beneficial to reduce the mean peak pressure	Park et al. (2014), Nouman et al. (2021), Shi et al. (2022)
	Nora Lunalastik EVA	Better reduction of peak contact pressure; Be a notable reduction of frictional stress under the first metatarsal head	
	Amfit	Be suitable for pressure offloading in the forefoot area	
Rigid material	Nora Lunalight A fresh	Rigid materials are less effective than soft materials in offloading plantar pressure	Nouman et al., 2019 (2021), Haris et al. (2021)
	Pe-Lite		
	TPU		
Combination of soft and rigid materials	EVA and TPU; Amfit and TPU	Have a beneficial effect on plantar pressure reduction and redistribution for a diabetic foot with neuropathy	Nouman et al. (2021)
Fabric	High porosity	Higher air and water vapor permeability than other materials	Rajan et al. (2016), Li et al. (2022)
New skin-like materials	Synthetic skin material; PVA/CMC	Higher flexibility and tensile strength than conventional materials	Chanda and Unnikrishnan (2018), Patwa et al. (2020)

Commonly used plastic, rubber, and silicone-based diabetic foot insoles are non-degradable, non-absorbent, and lack comfort due to their inclusion of magnetic protrusions (Ning et al., 2022). To address these shortcomings, researchers focus on developing new materials with high comfort and superior mechanical performance. Chanda and Unnikrishnan (2018) proposed a novel customized insole made of a synthetic skin material, demonstrating increased effectiveness in stress offloading at ulcers compared to conventional materials. Polyvinyl alcohol/carboxymethyl cellulose (PVA/CMC) based magnetic hydrogels exhibited improved flexibility and tensile strength over conventional materials, providing enhanced comfort (Patwa et al., 2020). Material choice significantly influences insole pressure-offloading capabilities and comfort (Lo et al., 2014; Park et al., 2014) found that materials with low-density structures and large foam cell sizes were suitable for direct contact with the foot, and Rajan et al. (2016) investigated air and water vapor permeability, as well as thermal properties, relative to the porosity of spacer fabric insoles. Porosity influenced air and water vapor permeability.

#### 3.1.3 Structure of the diabetic foot insole

The structure and material of diabetic foot insoles are closely intertwined. Single-layer insoles prioritize comfort, dual-layer insoles combine soft and rigid materials for both comfort and support, and multi-layered insoles use diverse materials for comprehensive support, control, and comfort. Different structures and materials yield varied effects on insole performance. Changing material alone may not simultaneously meet requirements for pressure offloading, comfort, and air permeability, necessitating structural optimization. Designing different porous structures within the insole can enhance pressure unloading and air permeability, offering adjustable parameters for personalized insoles. The internal structure of the insole also impacts the manufacturing process, constituting a crucial aspect of insole design.

Porous structures have gained traction in recent years for diabetic foot insole design. Tang et al. (2019) established relationships between equivalent modulus and structural parameters, enabling adjustable mechanical properties through various porous structure characteristics. Elliptical porous structural units, as proposed by Ma et al. (2019), demonstrated flexibility in adjusting geometric parameters and effective modulus for designing diabetic foot insoles (Shi et al., 2021). The honeycomb structure, employed for diabetic foot insoles, provides high stiffness and load-bearing capacity (Panda et al., 2018). Chen et al. (2021) developed an insole with a honeycomb and auxetic structure, effectively reducing pressure in the forefoot and rearfoot areas. Claybrook et al. (2022) suggested using a Split P TPMS structure to mimic medical-grade foams in diabetic foot orthoses, offering a range of compressive strengths by adjusting porosity. This structure can create a new generation of diabetic foot insoles adaptable to unique loading conditions. Compared to other porous structures, the elliptical porous structure and TPMS structure exhibit superior flexibility in adjusting geometric parameters, effective modulus, and porosity, facilitating personalized diabetic foot insole design. Table 3 summarizes representative porous structures.

**TABLE 3:** Summary of different types of porous structures.

Type of porous structures	Representative study	Main findings		Advantages and disadvantages	Refs
Elliptical porous structure	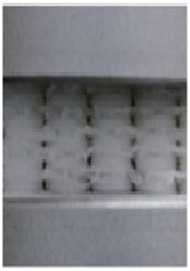	The elliptical porous structure meets the mechanical requirements of diabetic foot insoles		Flexibility in adjusting its geometric and mechanical parameters; Mechanical properties are influenced by eccentricity	Ma et al. (2019)
Honeycomb structure	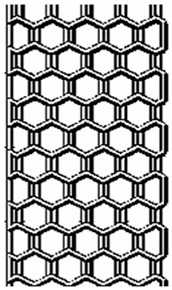	A novel insole with a honeycomb and auxetic structure can optimize stress distribution		High stiffness and can be used for carrying the load	Chen et al. (2021), Mondal et al. (2023)
Split P TPMS structures	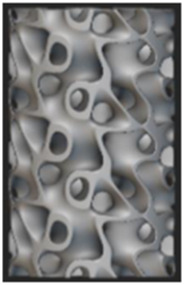	Split P TPMS structure could be used to mimic the properties of medical-grade foams for diabetic orthoses		A wider range of compressive strengths and cushioning afforded	Claybrook et al. (2022)

Given the unique foot shape and condition of each individual, standardized pressure offloading diabetic foot insoles may not suffice to meet every patient’s specific needs. Personalized insole design tailored to individual foot characteristics is crucial to minimizing the risks of pain and injury. Additionally, assessing other mechanical indicators, such as pressure gradient angle, pressure time integral, pressure mean value, and pressure fluctuation index (Lung et al., 2016), alongside peak plantar pressure, can offer a more comprehensive evaluation of diabetic foot in future research.

### 3.2 Design of smart monitoring diabetic foot insoles

Advancements in intelligent and informative technology have paved the way for combining emerging technologies with traditional medical interventions for diabetic foot prevention and management (Lung et al., 2020). Smart insole systems, a prominent technology in this domain (Majumder and Deen, 2019; Macdonald et al., 2021), monitor plantar pressure or temperature and transmit data to the user’s smartphone or watch via Bluetooth. Users receive alerts to reduce pressure by adjusting posture or gait, offering a convenient self-monitoring and prevention method (Lee et al., 2017; Macdonald et al., 2021).

**FIGURE 7:**
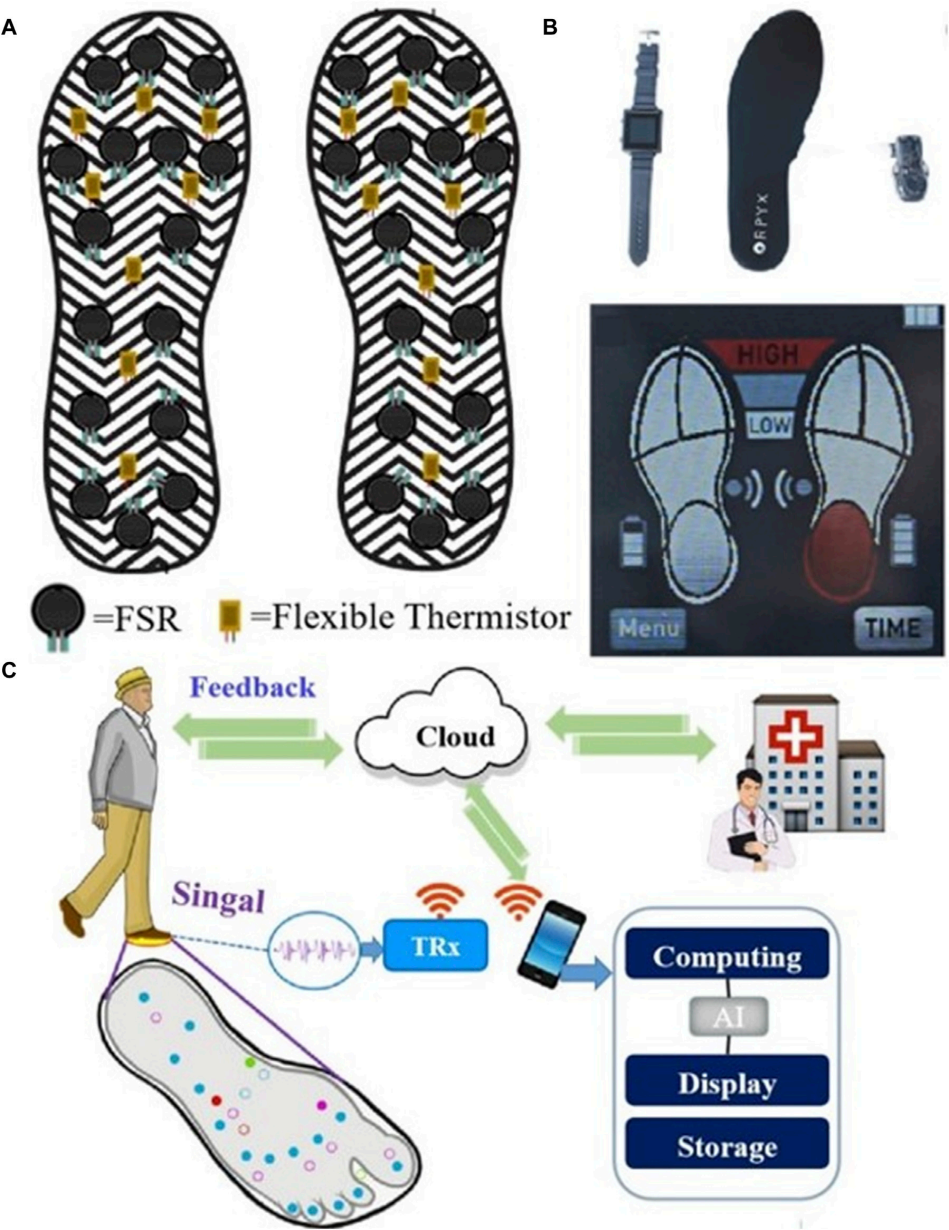
Smart monitoring insole. **(A)** The smart insole for monitoring plantar pressure and temperature with Force Sensitive Resistor (FSR) and Flexible Thermistor (Khandakar et al., 2022), **(B)** The sursense Rx smart insole system for monitoring plantar pressure (Abbott et al., 2019), and **(C)** An intelligent biofeedback system for older adults (Subramaniam et al., 2022).

Several studies have proposed smart insoles for monitoring dynamic plantar foot forces in diabetic patients to aid in preventing diabetic foot complications. Ha Van et al. (2017) introduced a wireless pressure-sensitive insole, FeetMe One, for plantar pressure monitoring, calculating plantar pressure maps and multiple gait patterns. Wang et al. (2022) proposed the portable Insole (SLIPS) system, equipped with 64 tri-axial force sensors, for continuous monitoring of plantar pressure and shear stress. In addition to pressure, temperature monitoring has garnered interest due to its association with an increased risk of ulceration in diabetic patients (Hernandez-Contreras et al., 2016; Martín-Vaquero et al., 2019; Gordon et al., 2020). Khandakar et al. (2022) presented an intelligent insole system that measured both plantar pressure and temperature (Figure 7A). This system enabled real-time monitoring of foot pressure and temperature, aiding in the early detection of foot problems. Yavuz et al. (2020) designed Temperature and Pressure Monitoring and Regulating Insoles (TAPMARI), emphasizing the combined benefits of temperature monitoring and plantar pressure offloading in preventing Diabetic Foot Ulcers (DFU). Addressing the high recurrence rate of DFUs with standard nursing methods, smart insole systems like SurroSense Rx (Orpyx Medical Technologies Inc., Calgary, Canada) have been employed (Figure 7B) (Najafi et al., 2017; Abbott et al., 2019). Abbott et al. (2019) demonstrated the efficacy of the SurroSense Rx system in reducing DFU recurrence by providing continuous feedback on plantar pressure and alerting users to adjust pressure throughout daily life via a smartwatch. Moreover, the integration of an intelligent biofeedback system and smart insoles contributes to the development of a telemedicine service system, efficiently diagnosing, managing, and treating patients based on various gait characteristics (Figure 7C). A compilation of research on intelligent monitoring of diabetic foot insoles is presented in Table 4.

**TABLE 4:** Key literature on different smart monitoring diabetic foot insoles.

Representative smart insoles	Monitoring data	Data feedback to users	Main findings	Refs
FeetMe One	Plantar pressure	Plantar pressure maps and multiple gait patterns	The maximum pressure threshold for these patients with the device was reduced to less than 80% of its previous level	Van Natten et al. (2020)
SLIPS System	Continuous plantar pressure and shear stress	Plantar pressure and shear stress maps	Comfortable (mean score = 4.3/5), caused some awareness of the insole (mean score = 2), did not impede natural gait (mean score = 4.3)	Wang et al. (2022)
SurroSense Rx	Continuous plantar pressure	Dynamic offloading guidance	The intervention group who wore the device had an 86% reduction in the incidence of ulcers	Abbott et al. (2019)
A Smart Insole System	Plantar pressure and temperature	Pressure map during different phases of the gait cycle; temperature Map during the stance position	The temperature measuring range is 20°C to 50°C; the generated maps can be used for early detection of diabetic foot complication	Khandakar et al. (2022)
TAPMARI	Temperature and pressure	Regulate foot temperatures at or below the target temperature	Providing approximately 4°C temperature relief between the contralateral feet (27.5°C vs. 31.6°C).	Yavuz et al. (2020)

Beyond monitoring pressure and temperature, it is crucial to consider the impact of humidity and acidity in diabetic foot care. Diabetic patients exhibit a diminished capacity to manage elevated heat and humidity compared to non-diabetic counterparts. The escalation of foot humidity during exercise or in higher ambient temperatures amplifies the risk of diabetic foot complications (Kularathne et al., 2019). Moreover, bacterial proliferation in ulcers can lead to increased wound acidity, contributing to the development of foot ulcers (Salvo et al., 2017b). Addressing these concerns, Kularathne et al. (2019) innovatively designed a device that seamlessly connects to any diabetic shoe, monitoring patients’ steps, weight, foot temperature, and humidity. In a parallel effort, Salvo et al. (2017a; Salvo et al., 2017b) developed a wearable sensor capable of tracking temperature and acidity changes in foot ulcers of diabetes patients over a week. Nevertheless, the existing literature lacks sufficient reporting on insoles designed explicitly for monitoring and mitigating foot humidity and acidity, underscoring the need for further research and evidence in this critical domain.

Despite the proven efficacy of intelligent monitoring in preventing and treating diabetic foot conditions, ensuring patient compliance is paramount. Prolonged wearing and continuous connection requirements for data uploading underscore the necessity for maintaining compliance to ensure accurate data collection and preserve the effectiveness of diagnosis and treatment.

## 4 Additive manufacturing of diabetic foot insoles

Traditional customized foot orthotics have historically been crafted through subtractive manufacturing processes, involving the carving or milling of materials to achieve the desired orthotic shape (Newman et al., 2015). This method, however, tends to be time-consuming, labor-intensive, and generates waste through excess material removal (Paris et al., 2016). In contrast, additively manufactured foot orthoses offer advantages such as high precision, customization, rapid production, and environmental sustainability. These attributes contribute to enhanced biomechanical control, comfort, risk reduction for pressure points, and shortened delivery times, ultimately expanding patient access to customized orthotics (Jin et al., 2015; Chen et al., 2016; Paris et al., 2016).

**FIGURE 8:**
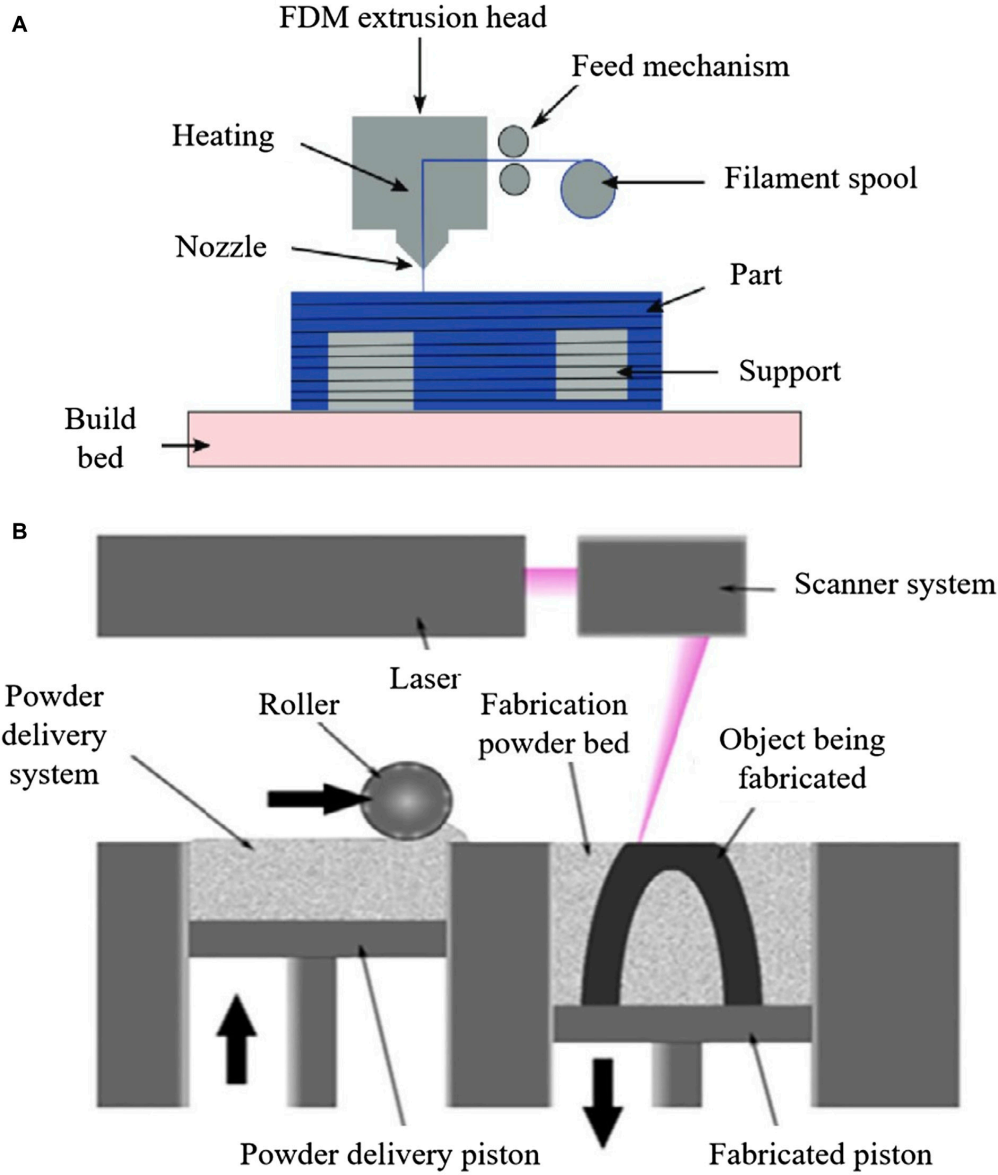
AM process for manufacturing diabetic foot insoles. **(A)** Illustration of the FDM process (Alafaghani et al., 2017). **(B)** Illustration of SLS Process (Abdulhameed et al., 2019).

As a burgeoning technology, additive manufacturing (AM) is gradually finding application in the production of diabetic foot insoles (Srivastava and Gaur, 2020). AM constructs three-dimensional objects by incrementally adding thin layers of materials guided by digital models (Wong and Hernandez, 2012). Its adoption streamlines production processes with short development cycles, and it is extensively utilized in the biomedical field (DebRoy et al., 2018). There are various additive manufacturing (AM) technologies, encompassing Selective Laser Sintering (SLS), Selective Laser Melting (SLM), Stereolithography (SLA), Fused Deposition Modeling (FDM), and 3D Printing (3DP) (Chen et al., 2016; Abdulhameed et al., 2019). Notably, FDM and SLS predominantly manufacture customized insoles for diabetic foot patients, as depicted in Figures 8A, B. FDM is the most commonly used technology for crafting customized insoles for diabetic feet, creating three-dimensional structures by depositing thermoplastic material onto a substrate in layers through a temperature-controlled printhead (Winarso et al., 2022). Research by Davia-Aracil et al. (2018) comparing traditional subtractive techniques to FDM for manufacturing anatomical insoles found FDM to be 43% more cost-efficient and time-effective. Ma et al. (2019) employed material extrusion techniques, including FDM and fused filament fabrication (FFF), to print adjustable modulus porous structures for diabetic foot insoles, demonstrating their mechanical effectiveness. Peker et al. (2020) implemented an upgraded FDM system to manufacture diabetic foot insoles with reduced production time.

While FDM is the most economical technique for creating entirely geometric customized insoles (Vyavahare et al., 2019), it has limitations. Interlayer bonding characteristics in FDM result in printed parts with highly anisotropic mechanical properties, potentially leading to early failure (Rankouhi et al., 2016; Lim et al., 2021). Additionally, materials used in FDM may exhibit suboptimal durability and mechanical properties (Hudak et al., 2022).

Another AM technology used for customized diabetic foot insoles is SLS, an industrial 3D printing method utilizing a powder bed to construct 3D objects (Fina et al., 2017). SLS involves selectively melting a thin layer of powdered material on a build platform according to a 3D model using a laser. This process is repeated layer by layer until the final object is formed (Mazzoli, 2013), allowing the production of complex structures with superior dimensional accuracy and strength (Awad et al., 2020). Li et al. (2022) highlighted SLS’s ability to successfully fabricate the unit array design beneath the insole’s lower surface. In comparison to FDM, SLS exhibits greater potential for manufacturing insoles with intricate structures. Table 5 provides a comparative overview of AM methods for diabetic foot insoles.

**TABLE 5:** Comparison of the AM methods for diabetic foot insoles.

AM method	Representative study	Main findings	Advantages and disadvantages	Refs
Fused Deposition Modeling (FDM)	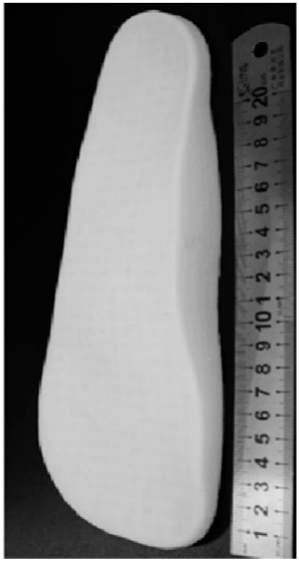	FDM is commonly used to make customized insoles for diabetic foot	Advantage: Cost-effective; wide range of thermoplastic materials available Disadvantages: High build time cost; low dimensional accuracy and resolution; limitations in fabricating complex structures	Ma et al. (2019), Peker et al. (2020), Kumar et al. (2022)
Selective Laser Sintering (SLS)	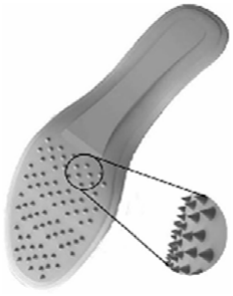	SLS has higher precision and the future application potential in the manufacture of diabetic foot insoles	Advantage: Be able to manufacture complex structures; varied materials available Disadvantages: Porous and mechanical weak in metal sintering components	Salles and Gyi (2012), Li et al. (2020), Sharma et al. (2020)

## 5 Performance evaluation of diabetic foot insoles

The evaluation of diabetic foot insoles encompasses both objective and subjective assessments. Objectively, evaluations focus on plantar pressure distribution analysis, gait analysis, and electromyography, primarily examining the pressure offloading performance of diabetic foot insoles (Bellomo et al., 2022). Prior to clinical application, a crucial step involves the performance evaluation of customized insoles for diabetic feet, ensuring accurate and efficient feedback to enhance the design (Anderson et al., 2020; Song et al., 2022).

The *in silico* method, also known as the numerical method, employs computational models to numerically evaluate a product’s performance (Anderson et al., 2021). Once developed and validated, it efficiently assesses the pressure-offloading performance of customized insoles (Peng et al., 2021). Unlike conventional experiments that involve human participants, *in silico* approaches, such as FE modeling, offer a more precise alternative by isolating the effects of specific insole design variables without the need for physical experiments on human subjects or models (Behforootan et al., 2017). However, the current challenge lies in developing valid computational models. Musculoskeletal models of the foot and foot-insole FE models have become common computational tools for evaluating diabetic foot insoles (Keenan et al., 2022). The musculoskeletal model is crafted to emulate human movement by integrating rigid bones, muscle fibers, tendons, ligaments, and other connective tissues crucial for understanding and predicting human activities (Falisse et al., 2019). Utilizing these models allows for an assessment of how insole design parameters impact everyday gait. Chen et al. (2015) introduced a 3D FE musculoskeletal model of the foot, providing a tool to evaluate the pressure-offloading performance of insoles made from different materials and thicknesses. Similarly, Jafarzadeh et al. (2021) employed a foot-insole FE model to scrutinize the influence of insole stiffness on shape optimization outcomes. Despite their utility, developing musculoskeletal models remains challenging in the current stage of research. These models are simplified representations, and as such, the boundary conditions derived from them may lack accuracy and appropriateness. The complexity of accurately capturing the intricacies of human biomechanics poses ongoing challenges in refining musculoskeletal models for robust and reliable evaluations of insole designs.

The *in vitro* evaluation method employs laboratory testing to assess the performance of diabetic foot insoles (Zhang et al., 2019). This testing utilizes a pressure measurement system to gauge the plantar pressure of subjects during both gait and standing (Burns et al., 2019; Anzai et al., 2022). The experimental results are then compared with FE simulation results to evaluate the pressure-offloading performance of the insoles. In its early stages, the F-Scan system served as the primary pressure measurement system; however, it encountered issues of poor accuracy and repeatability (Lorkowski et al., 2021). Subsequently, with advancements in science and technology, the Pedar system, renowned for its superior performance, has become widely adopted for pressure measurement (Yamamoto et al., 2020). Numerous researchers have conducted pressure tests on diabetic foot insoles using the Pedar system. Nouman et al. (2021), for instance, utilized the Pedar system to measure peak plantar pressure in diabetic foot patients while maintaining balance on level ground. The experimental average peak plantar pressure was then compared with the peak contact pressure obtained from FEA for validation. Tang et al. (2019) employed the Pedar-x insole pressure measurement system (Novel Pedar System, Germany) to measure contact pressure between feet and insoles during static standing with arms akimbo, assessing the insoles’ performance. D’Amico et al. (2021) utilized the Pedar-X system to measure in-shoe dynamic plantar pressure during walking in multiple trials along a 15 m walkway in a laboratory setting. Shi et al. (2022) obtained pressure values using the Pedar insole measurement system (Novel GmbH, Munich, Germany) to evaluate four different insoles during walking. Participants self-selected walking speeds to exhibit their typical gait characteristics on a 10 m straight sidewalk while barefoot and wearing insoles. A summary of *in vitro* performance evaluations of diabetic foot insoles is presented in Table 6.

**TABLE 6:** Key literature on *in vitro* performance evaluation of diabetic foot insoles.

AM method	Representative study	Main findings	Advantages and disadvantages	Refs
Early stage (approximately before 2010)	F-scan system (Tekscan Inc., Boston, USA) 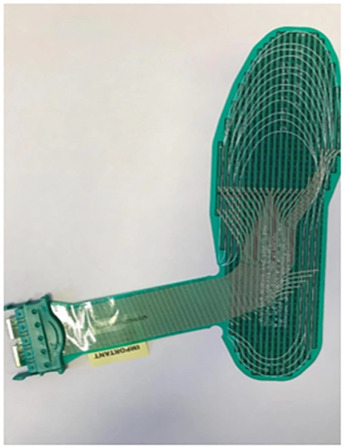	Walking at a self-selected and comfortable pace	Advantages: Low cost; thin in-shoe sensors; can be trimmed to fit shoe size and shape Disadvantages: Poor accuracy and repeatability Wearing the F-scan system changes gait characteristics	Cheung and Zhang (2008), Chatwin et al. (2020)
Nowadays (approximately 2010 to present)	Pedar-X (Novel Pedar System, Germany) 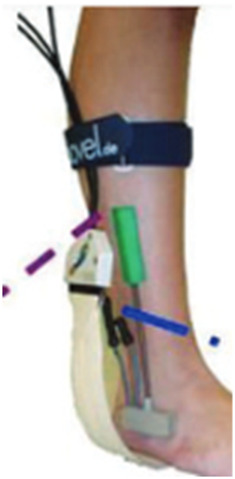	Standing in balance on level ground or still with arms akimbo	Advantages: High accuracy and repeatability; Effective for long-term vertical force measurement Disadvantages: Less accurate when low pressure is applied for short periods	Aliberti et al. (2011), Shi et al. (2022)

Subjective evaluation primarily involves collecting patient feedback on comfort, compliance, and overall satisfaction, typically obtained through randomized trials and questionnaires. Lavery et al. (2012) conducted a randomized trial with 299 patients, assessing standard care versus reduced insoles over 18 months. Patient questionnaires revealed insights into compliance and durability, impacting the effectiveness of shear-reducing insoles in preventing diabetic foot ulcers. In another study, Ming et al. (2019) conducted a 24-month trial evaluating sensor-equipped insoles for measuring foot temperature in 300 participants with diabetes and severe peripheral neuropathy. While objective measurements offer quantitative data, they may require specialized equipment or expertise. In contrast, subjective measurements provide qualitative data and may be more manageable. Combining both approaches could yield a more comprehensive assessment of diabetic insole effectiveness. A comparison of the advantages and disadvantages of each evaluation method is detailed in Table 7.

**TABLE 7:** Comparison of advantages and disadvantages of each evaluation method.

Evaluation method		Advantages	Disadvantages	Refs
Objective evaluation	Pressure Analysis	*In silico*: Visually display the plantar pressure distribution	The FE model is a simplified model, and its accuracy needs to be improved	Anderson et al. (2021), Peng et al. (2021)
		*In vitro*: Quantitatively evaluate the change of the plantar pressure	The walking conditions in the laboratory and daily life are quite different	Zhang et al. (2019)
	Gait Analysis	Determining the effect of diabetic foot insoles on foot biomechanical characteristics	Requires expensive instruments and specialized technicians	Burns et al. (2019), Anzai et al. (2022)
Subjective evaluation	Patient feedback	Reflect the patients’ experience in everyday life	Highly subjective; be affected by many factors, such as pain and emotion	Lavery et al. (2012), Abbott et al. (2019), Ming et al. (2019)

## 6 Challenges and future perspectives

In summary, customized insoles for diabetic individuals exhibit significant potential in effectively offloading plantar pressure, contributing substantially to early prevention, pain reduction, and economic burden alleviation. The continuous evolution of smart insoles for diabetic foot care is paving the way for an improved remote digital healthcare system (Ahmed et al., 2020). The ongoing advancement of smart insoles for diabetic foot care is paving the path toward an enhanced remote digital healthcare system. Nonetheless, despite the relatively comprehensive research on diabetic foot insoles, numerous limitations still require further exploration. Consequently, existing research encounters several challenges, and the following issues are underscored, along with corresponding suggestions for the design and implementation of diabetic foot insoles.

(1) Biomechanical Correspondence: Future studies must comprehensively consider the biomechanical correspondence between various tissues in the human foot, including bones, muscles, and fascia (Allan et al., 2016; Asghar and Naaz, 2022). Evaluating joint angles, muscle activity, and skeletal stress and strain is crucial for effective studies on diabetic foot, offering insights into foot motion, stability, muscle coordination, functionality, and predicting disease risks.
(2) Personalization in Design: Designing diabetic foot insoles should prioritize personalization in future studies (Lorkowski et al., 2021). While current research often bases designs on typical foot structures, the efficiency of insoles can be highly individualized due to differences in foot structure, arch type, or disease characteristics. Comprehensive consideration of the foot characteristics and conditions of diabetic patients is essential in the design and manufacturing process (Chen et al., 2016; Chhikara et al., 2022; Jin et al., 2015).
(3) Expanded Mechanical Indicators: In addition to peak pressure and shear force, future evaluations of diabetic foot should incorporate various mechanical indicators, including peak pressure gradient, pressure-time integral, pressure mean, pressure fluctuation index, foot skeletal stress, and contact area (Lung et al., 2016). These indicators offer a more comprehensive analysis, guiding the design of diabetic foot insoles effectively.
(4) Diversified Gait Simulation: To enhance the relevance of findings, future studies should expand the diversity of gait simulations. While many studies focus on neutral stationary stances and normal walking gaits, daily life involves varied and changing gaits (Nouman et al., 2019). Accounting for this diversity will reduce errors in pressure analysis results, aligning them more closely with actual plantar pressure distribution.
(5) Improved Patient Adherence: Enhancing patient adherence is crucial for the effectiveness of pressure-offloading insoles (Park et al., 2014). Adequate training on innovative insole technology is necessary to achieve the expected pressure-offloading effect of intelligent insoles, emphasizing the need for comprehensive patient education and support.

Based on the challenges highlighted earlier, several future research directions for the advancement of diabetic foot insoles can be outlined. These include:

(1) Reverse Design with Machine Learning: Utilizing machine learning for reverse design of insoles tailored to different levels of Diabetic Foot Ulcers (DFU). This involves exploring data characteristics, model training for parameter optimization, and selecting optimal insole designs, significantly reducing the overall design cycle.
(2) Topology Optimization with 3D Printing: Combining topology optimization technology with 3D printing to create lightweight insoles with guaranteed mechanical properties (Davia-Aracil et al., 2018; Awad et al., 2020). This approach involves designing personalized insoles with a dot matrix structure to optimize pressure distribution under the foot.
(3) Integration of Flexible Sensors: Integrating flexible sensors into smart insoles, utilizing sensors made of flexible materials that offer good flexibility, elasticity, and bendability (Cheung and Zhang, 2008; Chatwin et al., 2020). Flexible resistive pressure sensors can capture real-time information on plantar pressure changes, contributing to the development of intelligent and comfortable diabetic foot insoles.
(4) Monitoring Foot Humidity: Considering the monitoring of foot humidity for diabetic patients. This is particularly relevant given the reduced ability of individuals with diabetes to cope with high heat and humidity. Monitoring foot humidity can provide valuable information for reducing the risk of diabetic foot development (Ning et al., 2022). Despite this importance, there is a notable gap in devices reported in existing literature for addressing foot humidity concerns.

## 7 Conclusion

This review has provided a comprehensive overview of the design, manufacturing, and performance evaluation of diabetic foot insoles. The design phase often employs iterative optimization methods rooted in biomechanics and FEA, allowing for the prediction of design parameters associated with insoles that offer superior pressure offloading effects. The discourse also delves into the evolving landscape of smart insoles designed specifically for diabetic foot care. In the realm of manufacturing, while Fused Deposition Modeling (FDM) stands out for its cost-effectiveness and efficiency, its limitations in printing complex structures necessitate the use of Selective Laser Sintering (SLS) for intricate insole designs. Insoles featuring total contact customization, softer and absorbent materials, elliptical porous structures, or TPMS structures are deemed more fitting for diabetic foot prevention, taking into account both pressure offloading performance and comfort. Prior to clinical application, a thorough evaluation of insole performance through *in silico* and *in vitro* testing approaches is crucial. Despite the strides made in exploring various facets of diabetic foot insoles, there are avenues for further research. Future investigations could explore inverse design strategies leveraging machine learning, use topology optimization techniques for lightweight insole development, integrate flexible sensors for enhanced functionality, and delve into the development of novel skin-like materials tailored specifically for diabetic foot insoles. These advancements hold promise for refining the efficacy, personalization, and innovation of diabetic foot care technologies.
